# Distinct, ecotype-specific genome and proteome signatures in the marine cyanobacteria *Prochlorococcus*

**DOI:** 10.1186/1471-2164-11-103

**Published:** 2010-02-10

**Authors:** Sandip Paul, Anirban Dutta, Sumit K Bag, Sabyasachi Das, Chitra Dutta

**Affiliations:** 1 Structural Biology & Bioinformatics Division, Indian Institute of Chemical Biology, 4, Raja S. C. Mullick Road, Kolkata - 700 032, India; 2 Bioinformatics Centre, Indian Institute of Chemical Biology, 4, Raja S. C. Mullick Road, Kolkata - 700 032, India; 3 Present address: Plant Molecular Biology & Genetic Engineering Division, National Botanical Research Institute, Rana Pratap Marg, Lucknow - 226001, India; 4 Present address: Department of Pathology and Laboratory Medicine, Emory Vaccine Center, School of Medicine, Emory University, Atlanta, GA 30322, USA

## Abstract

**Background:**

The marine cyanobacterium *Prochlorococcus marinus*, having multiple ecotypes of distinct genotypic/phenotypic traits and being the first documented example of genome shrinkage in free-living organisms, offers an ideal system for studying niche-driven molecular micro-diversity in closely related microbes. The present study, through an extensive comparative analysis of various genomic/proteomic features of 6 high light (HL) and 6 low light (LL) adapted strains, makes an attempt to identify molecular determinants associated with their vertical niche partitioning.

**Results:**

Pronounced strand-specific asymmetry in synonymous codon usage is observed exclusively in LL strains. Distinct dinucleotide abundance profiles are exhibited by 2 LL strains with larger genomes and G+C-content ≈ 50% (group LLa), 4 LL strains having reduced genomes and G+C-content ≈ 35-37% (group LLb), and 6 HL strains. Taking into account the emergence of LLa, LLb and HL strains (based on 16S rRNA phylogeny), a gradual increase in average aromaticity, pI values and beta- & coil-forming propensities and a decrease in mean hydrophobicity, instability indices and helix-forming propensities of core proteins are observed. Greater variations in orthologous gene repertoire are found between LLa and LLb strains, while higher number of positively selected genes exist between LL and HL strains.

**Conclusion:**

Strains of different *Prochlorococcus *groups are characterized by distinct compositional, physicochemical and structural traits that are not mere remnants of a continuous genetic drift, but are potential outcomes of a grand scheme of niche-oriented stepwise diversification, that might have driven them chronologically towards greater stability/fidelity and invoked upon them a special ability to inhabit diverse oceanic environments.

## Background

Evolution of a microbe is often driven by its environment or life-style. Microorganisms adapted to some specialized environmental conditions have been reported to display conspicuous genome and/or proteome features [1-8]. Species of widely varying taxonomic origins, but thriving in same/similar environmental conditions such as high temperature or high salinity, may converge to similar genome and/or proteome composition. In contrast, closely related bacterial species inhabiting distinct ecological niches may display substantial genomic diversity [1-3,6,8-11]. Unveiling the plausible causes/consequences, at the genome and proteome levels, of such niche-dependent evolution of the microbial world poses a major challenge to the present-day life-scientists. The marine cyanobacterium *Prochlorococcus marinus *[12], having multiple ecotypes exhibiting distinct niche-specific phenotypic as well as genotypic characteristics, offers a useful system to address this issue.

*Prochlorococcus *are one of the most abundant life forms on this planet and more importantly, are major contributors to global photosynthesis [13-15]. A variety of *Prochlorococcus *strains, each specialized to dwell in different conditions of light, temperature and nutrient abundances [14,16-18] dominate the euphotic zones of the ocean - mostly between latitudes 40°S and 40°N, and sometimes beyond. To date, the complete genomes of 12 different strains of *P. marinus *have been sequenced (listed in Table 1) and wide variations have been observed in genetic architectures, genome sizes and genomic G+C-content of these strains [19]. On the basis of vertical niche partitioning, these 12 strains are classified into two major *Prochlorococcus *ecotypes: high light adapted (HL) ecotype being most abundant in surface waters and low light adapted (LL) ecotype dominating deeper waters [19,20]. 6 of the sequenced strains have been identified to belong to the LL group and the other 6 have been found to survive at HL conditions [19]. The phenomenon of oceanic niche differentiation for *P. marinus *has been previously investigated into - and several inferences regarding the effects of light adaptation, nutrient availability and predator influence on their genome evolution and diversification has been arrived at [21-24]. However, the full expanse of ecologically relevant differences in genomic, physicochemical and physiological characteristics among these strains are yet to be explored. In the present study, we have attempted to identify novel niche-specific molecular signatures in the genome and proteome compositions of 12 different *Prochlorococcus *strains, and also investigated the adaptive strategies of different *Prochlorococcus *strains for their survival in diverse oceanic environments.

**Table 1:** General features of 12 *Prochlorococcus *strains under study

Organism	Accession no. (Ref_Seq)	Genome Size (Mb)	G+C- content (%)	Abbreviation
**Low light adapted strains**				
*P. marinus *str. MIT 9313	NC_005071.1	2.41	50.74	LL1
*P. marinus *str. MIT 9303	NC_008820.1	2.70	50.01	LL2
*P. marinus subsp. marinus *str.CCMP1375 (SS120)	NC_005042.1	1.75	36.44	LL3
*P. marinus *str. MIT 9211	NC_009976.1	1.70	37.01	LL4
*P. marinus *str. NATL1A	NC_008819.1	1.90	34.98	LL5
*P. marinus *str. NATL2A	NC_007335.1	1.84	35.12	LL6
**High light adapted strains**				
*P. marinus *str. AS9601	NC_008816.1	1.70	31.32	HL1
*P. marinus *str. MIT 9312	NC_007577.1	1.71	31.21	HL2
*P. marinus subsp. pastoris *str.CCMP1986 (MED 4)	NC_005072.1	1.70	30.73	HL3
*P. marinus *str. MIT 9515	NC_008817.1	1.70	30.79	HL4
*P. marinus *str. MIT 9215	NC_009840.1	1.70	31.15	HL5
*P. marinus *str. MIT 9301	NC_009091.1	1.60	31.34	HL6

It is worth mentioning in this context that *Prochlorococcus *is the first documented example of genome shrinkage along with A+T enrichment in a free-living organism [25]. Earlier examples of genome reduction had been restricted to endosymbionts or pathogens with a host-dependent lifestyle, which evolve under the constraint of frequent population bottlenecks with a subsequent increase in genetic drift [2,3,26-29]. Considering the abundance of *P. marinus *in the marine ecosystem, their reductive genome evolution might not be influenced by similar population bottlenecks and resulting genetic drifts, and thus seems to be a more complex phenomenon to explain. Although *P. marinus *genome evolution has been investigated previously [25,30] and the event of genome shrinkage have been ascribed to various factors related to their growth in oligotrophic waters [20,23,31], selection for metabolic economy [25,31,32], loss of low fitness genes [33], and smaller cell sizes [25], it is still unclear to what extent it has been driven by any random genetic drift and/or other specific selection force(s). Our analyses indicate that the ecotype-specific molecular signatures exhibited by *P. marinus *strains under study are not mere remnants of a continuous genetic drift, but a potential outcome of niche-oriented stepwise diversification of *Prochlorococcus*, orchestrated by an array of interplaying adaptive forces.

## Results

In an attempt to understand the trends in molecular evolution in *Prochlorococcus*, we have analyzed various genome and proteome characteristics of 6 LL and 6 HL strains of *P. marinus*. The analyses of genome/proteome in *P. marinus *include the study of trends in codon, dinucleotide and amino acid usages, gene synteny of orthologous sequences, intergenic sequence composition, physicochemical properties of the encoded proteins and the extent of positive selection among different strains. These analyses were primarily directed towards the identification of niche-specific variations within different *Prochlorococcus *strains.

### Strand-specific asymmetry in synonymous codon usage in low light adapted *P. marinus *genomes

**Figure 1:**
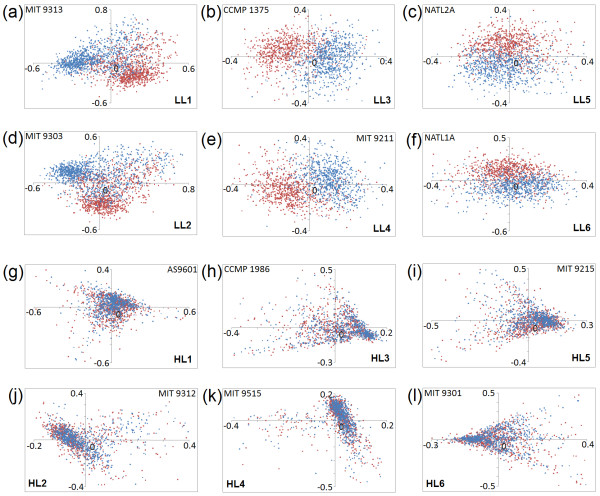
**Position of genes on the planes defined by the first (horizontal Axis1) and second (vertical Axis2) major axes generated by COA on RSCU values of coding sequences for each of the 12 *Prochlorococcus *strains (a to l)**. Genes transcribed from the leading and lagging strands are represented by red and blue coloured dots respectively.

In order to find out the trends in synonymous codon usage, we have carried out correspondence analysis (COA) on relative synonymous codon usage (RSCU) of 12 different strains of *P. marinus*. Figure 1 shows the positions of the individual genes on the planes defined by the first and second major axes generated by COA on RSCU values of coding sequences of respective *Prochlorococcus *genomes under study. Interestingly, in cases of all LL strains, the genes transcribed from the leading and the lagging strands of replication are segregated in two distinguishable clusters (with little overlap between them), either along Axis1 or Axis2 or both. Similar scatter plots with two distinct clusters of genes were observed earlier in cases of microbial genomes with pronounced strand-specific mutational bias [2,3,34,35]. When the positions of all synonymous codons are plotted on the plane defined by the first and second major axis of COA on RSCU for genes of all LL strains (plot not shown), a clear separation between G-/U- ending codons and A-/C- ending codons is observed, indicating the presence of asymmetric mutational bias at synonymous codon usage level in low light (LL) adapted genomes of *P. marinus*. In contrast, for each of the HL strains, a single cluster of genes (*i.e*., no segregation between the leading and lagging strand genes) is found on the Axis1-Axis2 plane of COA on their RSCU values (Figure 1), suggesting the absence of any pronounced strand-specific asymmetry in their synonymous codon usage. For all LL strains, GT3-content of genes exhibit significant correlations with Axis1 and/or Axis2 values (Table 2). 3 of the 6 LL strains, *viz*. LL1, LL3 and LL4 show highly significant correlations between Axis1 and GT3-content of genes, whereas LL2, LL5 and LL6 strains show significantly high correlations between Axis2 and GT3-content (Table 2). In LL strains the positions of the leading and lagging strand genes on the planes defined by Axis1 and Axis2 of COA (Figure 1), therefore, clearly indicate the overall GT3-richness of the leading strand genes. However, for the HL strains of *Prochlorococcus*, the percentage of variance explained by the first two principal axes are relatively low and the correlation values of these axes with GC3- or GT3-contents of genes are either insignificant or much lower than those observed for LL strains (Table 2). This suggests that no single axis and/or parameter can explain strand-specific variations in synonymous codon usage of HL strains.

**Table 2:** General features and correlations of GC3 and GT3 content with first two axes of COA on RSCU values of genes in 12 *Prochlorococcus *genomes

Organism		ORFs under study	G+C- content (%)	% of total variation explained by COA on RSCU		Correlation coefficient (r)			
				
						Axis 1 vs.		Axis 2 vs.	
						
				Axis 1	Axis 2	GC3	GT3	GC3	GT3
***P. marinus *str. MIT 9313**	**(LL1)**	1947	52.1	14.33	10.89	**-0.59**	**0.85**	**-0.69**	**-0.45**
***P. marinus *str. MIT 9303**	**(LL2)**	2080	51.6	15.93	12.29	**-0.89**	**0.43**	**-0.26**	**-0.86**
***P. marinus subsp. marinus *str. CCMP1375**	**(LL3)**	1492	37.1	7.47	6.23	**-0.15**	**-0.81**	**-0.58**	**0.25**
***P. marinus *str. MIT 9211**	**(LL4)**	1486	38.6	7.31	6.10	**-0.24**	**-0.78**	**0.52**	**-0.36**
***P. marinus *str. NATL1A**	**(LL5)**	1541	35.8	6.73	5.67	**-0.50**	**-0.24**	**-0.20**	**0.71**
***P. marinus *str. NATL2A**	**(LL6)**	1517	35.9	6.81	5.80	**0.54**	0.07	-0.10	**0.71**
									
***P. marinus *str. AS9601**	**(HL1)**	1463	31.9	5.51	5.07	-0.07	0.06	0.11	**-0.13**
***P. marinus *str. MIT 9312**	**(HL2)**	1503	31.8	5.85	5.20	**0.23**	-0.09	-0.09	0.10
***P. marinus subsp. pastoris *str. CCMP1986**	**(HL3)**	1451	31.5	5.96	5.68	-0.05	-0.06	**0.17**	0.03
***P. marinus *str. MIT 9515**	**(HL4)**	1465	31.6	5.78	5.49	0.11	-0.12	**-0.26**	0.00
***P. marinus *str. MIT 9215**	**(HL5)**	1506	31.8	5.67	5.25	**-0.21**	-0.07	0.09	**-0.14**
***P. marinus *str. MIT 9301**	**(HL6)**	1469	32.0	5.54	5.12	0.09	0.00	-0.13	-0.11

Correlations significant at p < 10^-6 ^are indicated in **boldface**.

Chi-square tests on occurrences of different codons on two replicating strands of representative LL strains (LL1 and LL6) further reveal significant overrepresentation of 28 and 22 G-/U- ending codons on the leading strands of LL1 and LL6 respectively (p < 0.001); while 28 and 25 A-/C- ending codons are overrepresented in the genes encoded on the lagging strands of LL1 and LL6 respectively (p < 0.001) (Additional files 1 and 2). The codon 'CUG' is the only exception, which, in spite of being G-ending, is significantly overrepresented in the lagging strand genes of LL1.

In microbial genomes characterized by pronounced strand asymmetry [2,3,34,35], replicational-transcriptional selection usually play a major role in shaping genome organization. The leading strands of replication of such organisms, in general, contain higher number of genes due to replicational selection, and are also enriched with highly expressed genes as an effect of transcriptional selection. However, in LL strains of *P. marinus*, the predicted protein coding sequences are found to be distributed almost equally in two strands. In fact, in three LL strains (LL1, LL2 and LL3), the number of predicted protein coding sequences are lower in the leading strands than in the lagging strands (approximately 48% in the leading strands and 52% in the lagging strands), indicating the absence of replicational selection. The lagging strands of the strains MIT9313 (LL1) and MIT9303 (LL2) are also found to be enriched in ribosomal proteins, which are typically highly expressed. Only two such genes are encoded by each of their leading strands, leaving 36 and 37 ribosomal proteins to be encoded by their lagging strands (LL1 and LL2 respectively). For other LL strains, the distribution of ribosomal genes is quite conventional (≈ 25 on leading strands and ≈ 12 on lagging strands). However, most of the other potentially highly expressed genes (*e.g*., RNA polymerases, transcription and translation processing factors, etc.), are present in higher numbers in the leading strands of all LL strains. Hence it is difficult to arrive at any definite conclusion regarding the effects of transcriptional selection on the LL strains of *Prochlorococcus*.

### Larger extent of genomic rearrangements between small and large *P. marinus *genomes

**Figure 2:**
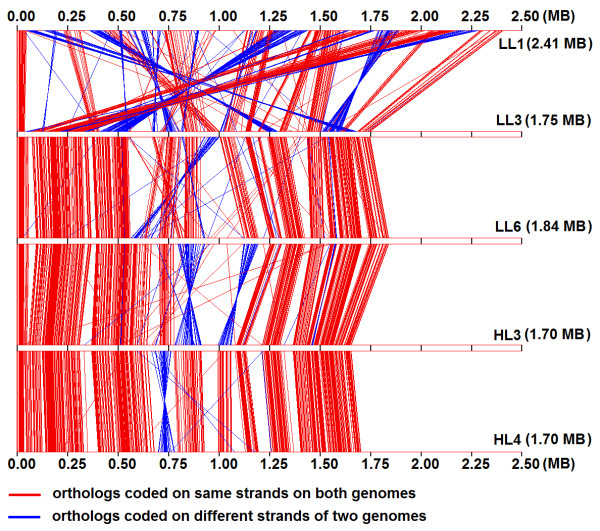
**Order of arrangement of 519 orthologs on chromosomes of 5 representative *Prochlorococcus *strains (LL1, LL3, LL6, HL3 and HL4)**. The red and blue lines join the locations of pair of orthologs on the linearly depicted chromosomes. Red lines indicate presence of the orthologous pairs on the same strand (either +/+ or -/-) of the two chromosomes they join, while the blue colour indicates the presence of the orthologs on different strands (+/- or -/+). Chromosomal scales are shown in megabase pairs (MB) and 0 MB represents the predicted origins of replication for the 5 strains.

In order to understand the nature and extent of genomic rearrangements during the events of niche specific diversification, we have analyzed the conservation of the relative order or synteny of orthologs across the chromosomes of different *P. marinus *strains. Figure 2 represents graphically, the gene synteny of 519 orthologs (see methods) in three low light (LL1, LL3 and LL6) and two high light adapted (HL3 and HL4) strains of *P. marinus*. The low light strains of *Prochlorococcus *have considerable variations in their genomic size and G+C-content (Table 1). The LL1 strain here represents the high G+C and large genome containing LL strains, while the LL3 & LL6 strains represent the other members of the LL group which have lower G+C-content and reduced genome size. HL3 and HL4 strains are representatives of the HL group, which also share smaller genome and lower G+C content. Interestingly, the extent of variation in order and orientation of orthologous genes between two strains of similar light optima but of distinct genome sizes and G+C-content (*viz*., between LL1 and LL3, Figure 2), is significantly greater than that between two strains with distinct light optima, but having similar genome size and G+C-content (*e.g*., between LL6 and HL3). It is worth mentioning at this point that this conclusion holds good for all 12 *P. marinus *strains under study, *i.e*., the results will be similar even if the representative strains selected here are replaced with any other strains form the groups they represent. The analysis, therefore, suggests that genome reduction in *P. marinus *has been accompanied by numerous seemingly random genome rearrangements such as translocations and inversions. Amongst the reduced genomes, the order and orientation of orthologous gene clusters remain more or less conserved except some local reorientation of genome fragments, the number and extent of reorientations being higher between the strains with distinct light adaptation (Figure 2).

### Niche-specific dinucleotide abundance values of *P. marinus *genomes

**Figure 3:**
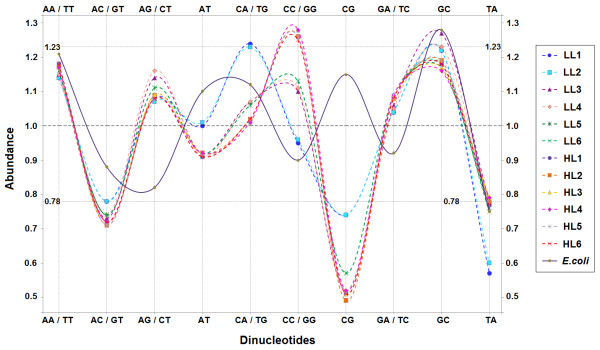
**Plot of dinucleotide abundance profiles of *E. coli *and 12 *Prochlorococcus *strains**. Differently coloured lines join the abundance values of dinucleotide pairs for each of the organisms. Abundance values ≥ 1.23 or ≤ 0.78 are significantly over or underrepresented (as described by Karlin *et al*., Theoretical population biology, 61, 367-390, 2002).

It has been reported previously that dinucleotide abundance values are usually similar in related species and can be regarded as a genome signature [36]. To understand the patterns in dinucleotide signature, we have calculated dinucleotide abundance values for all *P. marinus *genomes and also for *E. coli *(a representative outgroup). The genomic G+C-bias of *E. coli *is similar to that of LL1 and LL2 *Prochlorococcus *strains. The results are shown diagrammatically in Figure 3. Some *Prochlorococcus*-specific trends are exhibited by all *P. marinus *organisms under study, irrespective of their G+C-bias or ecological adaptation. For instance, the dinucleotide CG is appreciably overrepresented in *E. coli*, but significantly underrepresented in all *P. marinus*, including the strains which have similar G+C-content to that of *E. coli*. Contrasting trends in *E. coli *and *P. marinus *strains are also observed for the dinucleotides AG/CT and GA/TC. The dinucleotide abundance values of AC/GT are also significantly underrepresented in all *P. marinus *strains, but not in *E. coli*.

However, significant intra-*Prochlorococcus *differences are also present in dinucleotide abundance profiles, on the basis of which all *P. marinus *strains under study may be divided into three distinct groups:

(a) Group LLa, comprised of the two LL strains *P. marinus *MIT 9313 (LL1) and MIT 9303 (LL2) - both having larger genomes (≈ 2.5 MB) and average G+C-content ≈ 50%: Genomes of these two strains are characterized by significantly high values of CA/TG and low values of TA (Additional file 3). The values for AT, AC/GT and CG are also relatively higher and that of CC/GG are lower, as compared to other *P. marinus *strains.

(b) Group LLb, consisting of other four LL strains (LL3, LL4, LL5 and LL6), characterized by relatively lower G+C-content (between 35% - 37%) and small genome size (< 2 MB): These four LL strains exhibit highly similar patterns, which are visibly distinct mainly at CA/TG and CC/GG from the almost overlapping profile of HL strains (Figure 3).

(c) Group HL, including all 6 HL strains having reduced genome and G+C-content ≈ 31%: The dinucleotide CC/GG is significantly overrepresented only in the HL *Prochlorococcus *strains.

### Clustering by amino acid composition reveals a balance between genomic G+C-bias and *Prochlorococcus*-specific selection forces

In an attempt to investigate whether the strand-specific mutational bias has any impact on amino acid usage in gene products of LL strains of *P. marinus *in comparison to their HL counterparts, we performed correspondence analysis (COA) on relative amino acid usage (RAAU) of the encoded proteins of each organism. No clear segregation can be observed for proteins encoded by the leading and lagging strands in any of the *P. marinus *genomes under study (data not shown), implying that the strand-specific mutational bias has hardly any influence on the amino acid compositions of the gene products of LL strains of *Prochlorococcus*. In all the strains of *P. marinus*, the first three axes generated by COA on amino acid usage cumulatively explain about 39% of the total variability. Both mean hydrophobicity and aromaticity of the encoded proteins exhibit strong correlations with either of the first two principal axes and seem to be the major contributors to amino acid usage variation in *P. marinus *proteins (data not shown).

**Figure 4:**
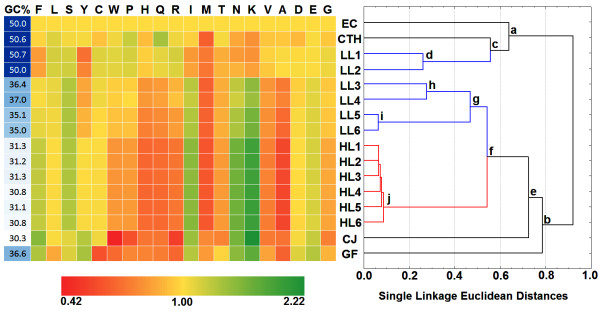
**Single linkage (Euclidean distances) clustering based on standardized amino acid usages (with respect to *E. coli*) of 12 *Prochlorococcus *strains and 4 other microbes, accompanied by a heatmap representation of the standardized amino acid usage values**. The overrepresentation or underrepresentation of amino acid residues in the organisms are shown in green and red colored blocks of varying colour intensities, respectively. [Abbreviations, EC → *E. coli*; CJ → *C. jejuni*; GF → *G. foresetii *and CTH → *Cyanothece sp*.]

In order to check whether the amino acid usage patterns in LL and HL groups of *Prochlorococcus *follow any specific trends, we carried out a clustering analysis on relative abundances (with respect to *E. coli*) of different amino acid residues of each organism (Figure 4) in a dataset comprising of all *P. marinus *strains under study along with a cyanobacterial representative *Cyanothece sp*. (having G+C-content similar to those of *E. coli *and two LLa strains), and two non-cyanobacterial species - the bacteroidetes/chlorobi *G. foresetii *(average genomic G+C-content = 36.6%, similar to those of LLb strains) and the epsilon-proteobacteria *C. jejuni *(average genomic G+C-content = 30.3%, similar to those of HL strains). Branching patterns suggest an optimization between the G+C-bias and *P. marinus *group-specific selection for amino acid usage. As can be seen in Figure 4, a major branching between the organisms occurs according to the average genomic G+C-content. Organisms having average G+C-content around 50% - *viz. E. coli*, *Cyanothece sp*. and the two LLa strains - cluster together under the node 'a', while the organisms with lower G+C-content (30-37%) form a distinct cluster at the node 'b'. However, within the A+T-rich or relatively G+C-rich clusters, finer segregation occur according to taxonomy, *i.e*., between the cyanobacterial and non-cyanobacterial species. For instance, node 'a' acts as a bifurcation point between the gamma-proteobacteria *E. coli *and the three cyanobacterial species, followed by another bifurcation at node 'c' between the two LLa strains and non-*Prochlorococcus *species *Cyanothece*. Similarly, within the A+T-rich cluster, the HL strains of *P. marinus *with G+C-content ≈ 30-31% club together with their LLb counterparts (average G+C-content ≈ 35-37%) under the node 'f', distinctly separated from *C. jejuni *and *G. foresetii*. Node 'f' also acts as the point of divergence of LLb and HL strains, which cluster separately under the nodes 'g' and 'j' (containing 4 LLb strains and 6 HL strains respectively). These observations indicate that though the amino acid usage patterns in *Prochlorococcus *are primarily guided by their directional mutational bias, other selection pressures must also have exerted some significant influence. Three cyanobacterial species, namely *Cyanothece sp*., *P. marinus *str. MIT9313 (LL1) and *P. marinus *str. MIT9303 (LL2) - all have nearly the same G+C-content as *E. coli*, yet their amino acid usage abundance patterns are quite distinct from that of *E. coli*. Similarly, the LLb and HL strains of *P. marinus *display significant differences in amino acid usage patterns from that of non- *P. marinus *species (*G. foresetii *and *C. jejuni*, respectively) having similar G+C-content. A careful examination of Figure 4 delineates the *Prochlorococcus*-specific features in amino acid usage patterns. For instance, as compared to *E. coli*, Thr is underrepresented in all strains of *P. marinus*, including those having ≈ 50% G+C-content (and also in *C. jejuni*, but not in *Cyanothece *and *G. forsetti*). Ser is overrepresented in all A+T-rich *P. marinus *species, especially in comparison to *C. jejuni *and *G. foresetii*. Tyr is typically underrepresented in all *Prochlorococcus *strains, compared to the non-*Prochlorococcus *species of similar G+C-content. Frequency of Phe is also relatively lower in *Prochlorococcus *in comparison to any other species of similar G+C-bias. All these observations suggest that the amino acid usage in *P. marinus *is a result orchestrated by the forces of species-specific selection acting on mutational bias and genetic drift.

### Niche-specific variations in physicochemical and structural features of *Prochlorococcus *orthologs

In an attempt to have a better insight into the niche-specific physicochemical and structural properties of *P. marinus *proteins, if any, we performed a comparative analysis of the core proteins, *i.e*., proteins found to be present in all *P. marinus *strains under study. Different proteomic properties of 519 orthologous proteins between all 12 *P. marinus *strains are summarized in Table 3, which shows a gradual increase in average aromaticity and pI values and decrease in mean hydrophobicity and instability indices of orthologs, as one moves from the members of group LLa to group LLb to group HL. In other words, the core proteins of LL strains are, in general, more hydrophobic, more acidic, less aromatic and less stable than their HL orthologs. The comparison of structural properties reveals that among the three groups, members of the group LLa exhibit the highest propensity for helix formation and lowest propensity for beta-sheet and coil formation. LLb group orthologs are characterized by intermediate values for all three propensities, and group HL orthologs display trends opposite to that of group LLa - *i.e*. lowest propensity for helix formation and highest propensity for beta-sheet/coil formation (Table 3).

**Table 3:** Different amino acid indices and secondary structural traits of 519 orthologous proteins present in 12 *Prochlorococcus *strains

**Organism**	**Amino acid indices (Mean)**				**Secondary structural traits (%)**		
		
	**Hydrophobicity**	**Aromaticity**	**Isoelectric Point (pI)**	**Instability Index (II)**	**Alpha helix**	**Beta sheet**	**Coil**
		
**LL1**	-0.11	0.067	6.62	40.61	37.51	13.76	48.73
**LL2**	-0.11	0.067	6.65	40.71	37.45	13.74	48.81
**LL3**	-0.16	0.072	7.07	38.88	35.85	14.58	49.57
**LL4**	-0.16	0.072	7.08	38.81	36.13	14.26	49.61
**LL5**	-0.18	0.073	7.08	38.09	35.15	14.68	50.17
**LL6**	-0.18	0.073	7.07	38.10	35.15	14.69	50.16
							
**HL1**	-0.20	0.077	7.15	37.03	34.32	15.33	50.35
**HL2**	-0.20	0.077	7.18	37.19	34.31	15.36	50.33
**HL3**	-0.20	0.077	7.23	37.33	34.42	15.36	50.22
**HL4**	-0.20	0.077	7.32	37.36	34.54	15.27	50.19
**HL5**	-0.20	0.077	7.21	37.02	34.38	15.27	50.35
**HL6**	-0.20	0.077	7.14	37.15	34.35	15.20	50.45

However, one may argue that these inter-group variations in physicochemical properties and structural propensities of *P. marinus *strains can only be a reflection of their varying genomic G+C-bias rather than being niche specific. In order to address this issue, we carried out a comparative analysis of various proteomic features of orthologous sequences from three representative *P. marinus *strains LL1, LL3 and HL3 (from groups LLa, LLb and HL respectively) with those of three other caynobacterial species, *Synechococcus elongatus *(55.5% G+C-content), *Synechocystis sp*.(47.4% G+C-content) and *Nostoc sp*.(41.3% G+C-content) as well as three non-cyanobacterial species namely *E. coli*, *Bacillus cereus *and *Francisella tularensis*. The non-cyanobacterial species were chosen as reference organisms, due to their close average genomic G+C-content to those of LLa, LLb and HL strains (50.8%, 35.5% and 32.3% for *E. coli*, *B. cereus *&*F. tularensis*, respectively), and the significant number of orthologs they share with these *P. marinus *strains. Values of different physicochemical parameters and structural propensities of the orthologs from these reference species and the representative *P. marinus *strains are summarized in the Table 4. It reveals that the values of any specific parameter, say of hydrophobicity index or instability index, is in most cases, not comparable between orthologs from *P. marinus *strains and outgroup organisms of similar G+C-bias. For instance, the values observed for *B. cereus *are quite different from those of LL3 - the genomic G+C-content of both being quite similar. The average pI value of *B. cereus *proteins is not only significantly less than that of their LL3 orthologs, it is even lesser than LL1 proteins. The average instability index of *B. cereus *proteins is also much less than that of the *Prochlorococcus *orthologs. The average helix forming propensity of *B. cereus *proteins is closer to that of LL1 proteins, while their beta sheet forming propensity is almost same to that of HL3 proteins. Similarly, the aromaticity and instability indices or helix forming propensities of *E. coli *proteins are significantly different from those of *Prochlorococcus *strain LL1, while most of the *F. tularensis *protein characteristics differ widely from those of the *P. marinus *HL3 strain with similar G+C-bias. Comparing between the cyanobacterial species, amino acid indices and secondary structural traits of *Synechococcus *(the closest taxonomic relative of *Prochlorococcus*) seem to be guided by its G+C-content (Table 4: Set IV). The hydrophobicity values and the helix-forming propensities of the other two cyanobacteria also gradually decrease with decrease in genomic G+C-content within the set. However, the other indices and structural traits of *Synechocystis *and *Nostoc *do not reflect any systematic co-variation with their G+C-bias. These observations suggest that the variations in proteomic features of *Prochlorococcus *might not be a mere outcome of their G+C-bias, there could be significant influence of other selection forces as well.

**Table 4:** Comparison between various amino acid indices and secondary structural traits of six sets of proteins of *Prochlorococcus *and non-*Prochlorococcus *orthologs

**Organisms**		**Mean of amino acid indices**				**Secondary structural traits (%)**		
			
		**Hydrophobicity**	**Aromaticity**	**pI**	**Instability Index**	**Alpha helix**	**Beta sheet**	**Coil**
		
**Set I (303 pairs)**	**BC**	-0.14	0.08	6.51	34.34	38.62	16.64	44.74
	**LL1**	-0.08	0.06	6.61	38.72	38.07	14.33	47.60
	**LL3**	-0.13	0.07	7.09	37.33	36.37	15.22	48.41
	**HL3**	-0.17	0.07	7.23	36.17	34.65	16.01	49.35
		
**Set II (136 pairs)**	**EC**	-0.05	0.08	6.91	36.58	40.26	14.43	45.31
	**LL1**	-0.07	0.07	6.57	38.94	37.44	14.76	47.80
	**LL3**	-0.12	0.07	7.13	37.65	35.59	16.17	48.24
	**HL3**	-0.15	0.08	7.30	35.63	33.87	16.63	49.49
		
**Set III (265 pairs)**	**FT**	-0.15	0.08	7.30	33.68	38.22	16.60	45.19
	**LL1**	-0.12	0.06	6.64	39.46	37.71	14.35	47.94
	**LL3**	-0.17	0.07	7.18	37.93	36.03	15.12	48.85
	**HL3**	-0.21	0.07	7.33	36.57	34.56	15.79	49.64
		
**Set IV (175 pairs)**	**SN**	-0.03	0.07	6.88	41.23	40.46	13.39	46.14
	**LL1**	-0.06	0.06	6.61	39.39	37.54	14.27	48.19
	**LL3**	-0.10	0.07	7.15	37.82	35.47	15.13	49.40
	**HL3**	-0.15	0.08	7.25	35.96	34.25	15.73	50.01
		
**Set V (963 pairs)**	**SSP**	-0.09	0.08	6.41	38.77	37.72	14.60	47.68
	**LL1**	-0.06	0.07	6.76	42.06	38.80	13.15	48.05
	**LL3**	-0.11	0.08	7.45	38.95	36.00	14.81	49.18
	**HL3**	-0.16	0.09	7.81	36.21	34.34	16.12	49.55
		
**Set VI (961 pairs)**	**NOS**	-0.08	0.08	6.58	38.59	38.35	15.05	46.61
	**LL1**	-0.06	0.07	6.81	41.96	39.00	13.11	47.89
	**LL3**	-0.11	0.08	7.49	38.87	36.25	14.74	49.01
	**HL3**	-0.17	0.09	7.82	36.30	34.27	16.09	49.62

**BC **- *Bacillus cereus *(35.5% G+C), **EC **- *Escherichia coli *(50.8% G+C), **FT **- *Francisella tularensis *(32.3% G+C), **SN **- *Synechococcus elongatus *(55.5% G+C), **SSP **- *Synechocystis sp*. (47.4% G+C), **NOS **- *Nostoc sp*. (41.3% G+C)

### Higher positive selection between orthologs from strains with opposite light optima

**Figure 5:**
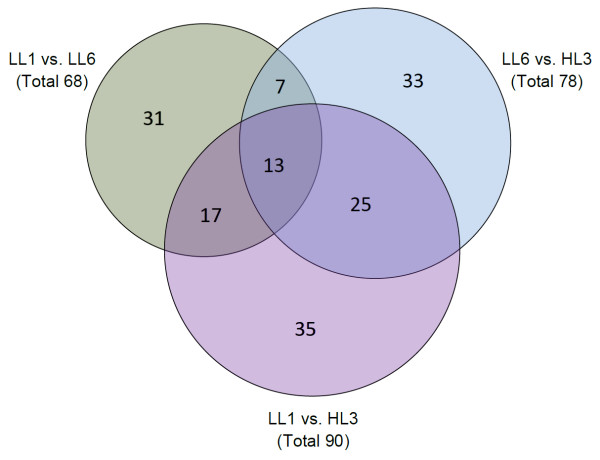
**Venn diagram depicting the number of positively selected (d_N_/d_S _> 1) orthologs between pairs of different *Prochlorococcus *strains**. The 519 core proteins of 3 representative organisms (LL1, LL6 & HL3) were considered for the analysis. d_N_/d_S _could be calculated for 414 × 3 pairs out of the 519 × 3 pairs considered.

In order to better understand the evolutionary trends in different *P. marinus *species having distinct genome composition and/or light adaptation, the rates of synonymous and non-synonymous substitutions (d_S _and d_N_) were calculated between 519 orthologous sequences of LL1, LL6 and HL3 strains (representatives of groups LLa, LLb and HL respectively), and the number of genes showing positive selection (d_N _> d_S_) between each possible pair of organisms were determined. Figure 5 depicts a Venn diagram for the number of positively selected genes among the strains under study. Out of 519 orthologs, maximum number of positively selected genes (90) is found between LL1 and HL3 - the strains that differ in genome size, G+C-composition and light adaptation. The strains LL6 and HL3 having nearly similar genome size and G+C-bias, but distinct light optima come next with 78 positively selected genes among them and the two strains LL1 and LL6 of the same light group, but distinct genome size and G+C-bias, exhibit the minimum number (68) of positively selected genes. Among these three sets, there are several genes, which are positively selected between any two out of the three possible pairs of *P. marinus *strains under study. There are 25 genes selected positively between HL3 and either of the LL strains *i.e*. between the strains of two opposite light optima, irrespective of their G+C-bias and genome size. 17 genes are positively selected between the strains of distinct G+C-bias and genome size - between the relatively G+C-rich and large genome strain LL1 and either of the A+T-rich and reduced genome strains LL6 or HL3. Only 7 genes display common positive selection between LL6 and LL1 (the strains with similar light adaptation but of different genome size and G+C-bias) and between LL6 and HL3 (the strains having relatively lower G+C-bias and genome size). Thus, our study indicates the presence of a considerable positive selection pressure in diversification of the *Prochlorococcus *core genome, which in turn, suggests an appreciable role of random genetic drift in vertical niche partitioning of the strains.

### Pronounced effects of directional mutational bias in the intergenic regions of HL *P. marinus *strains

In an attempt to examine whether the G+C-bias of intergenic regions of the different strains (with varying genomic G+C-content), follow trends similar to the respective coding regions, G+C-content of intergenic regions were calculated. The intergenic regions are, in general, more A+T rich than the overall genomic G+C-content of respective organisms (Additional file 4). Also, the A+T bias of intergenic regions are more pronounced in HL strains than their LL counterparts.

Unconstrained intergenic regions are more prone to mutational change. Accumulation of unfavourable mutations may render a coding region nonfunctional, facilitating its removal from the genome in course of time. We have identified probable remnants of coding sequences within intergenic regions of two representative *Prochlorococcus *strains having reduced genomes (LL6 and HL3). The G+C-content of these remnants are, in most cases, higher than that of average intergenic DNA, but lower than the average G+C-content of the *bona fide *coding regions (Table 5 and Additional file 5). These particular non-coding sequences, therefore, may be remnants of coding sequences that are in the process of being eliminated from the genome.

**Table 5:** Putative remnants of coding regions and their G+C-content (%)

**Organism**	**Putative remnants of coding regions**			**Overall G+C%**		
		
	**No. of hits**	**Average G+C%**	**Standard Deviation**	**Intergenic**	**Coding**	**Genomic**
		
LL6	48*	33.78	4.87	28.76	35.98	35.12
HL3	93*	26.35	4.04	23.33	31.64	30.80

* for details see Additional file 5

## Discussion

**Figure 6:**
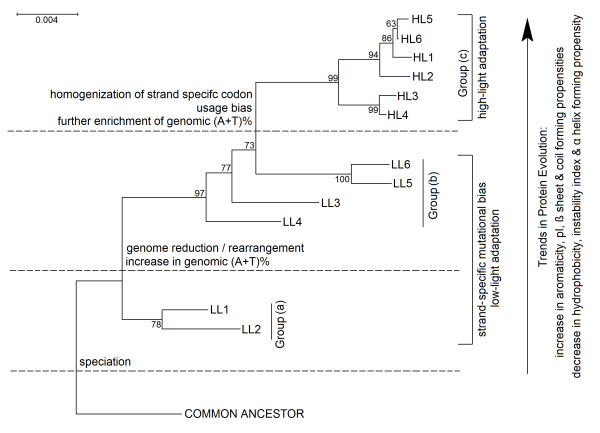
**Trends in genome/proteome evolution of *Prochlorococcus*, suggesting a stepwise diversification of the ecotypes**. The model is based on the 16S rRNA phylogeny of the 12 strains, inferred from a bootstrap consensus tree (500 replicates) generated using the Minimum Evolution method (CNI algorithm), with the software MEGA (version 4).

Exhibition of a wide range of genomic G+C-content (30.8% to 50.7%) and genome sizes (1.6 Mb to 2.7 Mb) by different strains of *P. marinus*, and also their adaptation to different ecological niches - a situation encountered rarely in the microbial world - demand detailed investigation. We have performed a large scale comprehensive study to critically analyze the direction and strength of mutational pressure and genomic/proteomic determinants associated with the adaptation of these strains to oceanic environments subject to different light intensities. From this study it appears that (a) low light adapted (LL) free living *Prochlorococcus *strains exclusively show strand asymmetry in synonymous codon usage, (b) general trends in amino acid usage in LLa, LLb and HL strains differ appreciably, (c) distinct dinucleotide abundance profiles are exhibited by LLa, LLb and HL strains, (d) higher number of genes have undergone positive selection between the strains with distinct light optima, *i.e*., between LL and HL strains and (e) there are definite trends in variations of different physicochemical and structural features in core proteomes of different groups of *Prochlorococcus *strains, which are not solely governed by their genomic G+C-bias. These observations, along with the findings on large-scale genome reduction associated with gradual increase in genomic A+T-content and extensive chromosomal rearrangements between different strains, strongly suggest a stepwise diversification of *Prochlorococcus *strains, in course of their adaptive evolution (Figure 6).

Among several genome/proteome signatures of *P. marinus *strains reported for the first time in this work, the most notable is the impact of pronounced replication-strand-specific asymmetry on synonymous codon usage, observed exclusively in the low light adapted strains of *P. marinus *(Figure 1). This is noteworthy for two reasons: (i) Presence of pronounced strand-specific mutational bias with detectable influence on codon usage was observed so far mostly for obligatory intracellular microorganisms having reduced genomes [2,3,5]. Interestingly, all 6 LL strains of *P. marinus *exhibiting strand-specific synonymous codon usage are free-living and two of them (LL1 and LL2) are characterized by relatively larger genome size. On the other hand, for the reduced genomes of 6 HL strains, no perceivable sign of strand asymmetry could be seen in their usage of synonymous codons. (ii) In most of the other microbial genomes with asymmetric mutational bias, the genes, especially the highly expressed ones, are present in the leading strands of replication in significantly higher numbers, the phenomenon referred to as replicational-transcriptional selection [2,3,34,35]. No such definite significant bias in gene distribution is observed in either of the strands of replication in the LL strains of *P. marinus*. Strand asymmetry in codon usage of *Prochlorococcus*, therefore, may not bear an explicit causality to the event of genome reduction or with replicational-transcriptional selection.

The homogenization of the strand asymmetric bias in the HL strains may be attributed, at least partially, to the absence of a specific type of DNA repair enzyme MutY. In previous studies of Rocap *et al*. [20] and Dufresne *et al*. [25] it have been shown that the enzyme MutY is absent in the strain *P. marinus *str. CCMP1986 (HL3), while it is present in *P. marinus *str. CCMP1375 (LL3) and *P. marinus *str. MIT9313 (LL1). MutY, an A/G-specific DNA glycosylase, acts with MutT (NTP pyrophosphohydrolase) and MutM (formamido-pyrimidine-DNA glycosylase) to avoid misincorporation of oxidized guanine (8-oxoG) in DNA and to repair the base mismatches A:8-oxoG [37]. Knocking out both *mutM *and *mutY *in *E. coli *results in a 1,000-fold increase of G:C to A:T transversions in comparison to the wild-type strain [38]. Our analysis reveals (through BLASTP search) that *mutY *is present only in the LL strains, but not in any of the 6 HL strains. The excess number of 'G's present in the leading strands of LL strains might have transversed to 'A's in the HL strains due to the absence of *mutY *in the later, and this in turn, caused a simultaneous increase of 'T's in the lagging strands, eventually leading to homogenization of the G+T and A+C frequencies in two strands of replication in the HL strains. Existing mutational drift towards A+T-enrichment in the HL strains might also have facilitated achieving the uniformity in those strains. Further insights may be accumulated in this regard with the availability of more completely sequenced *Prochlorococcus *genomes in future.

In the process of gradual genome reduction, mutations often accumulate in expendable genes, thereby transforming them, by degrees, to pseudogenes, to small fragments, to extinction [39]. In the reduced genomes of *P. marinus*, we have found some putative remnants of coding regions, the A+T-content of which are, in general, higher than that of coding regions, but lower than other non-coding regions. This is in agreement with the fact that the reduced genomes of *P. marinus *(especially those of HL strains) are subject to a strong mutational A+T-drift, and will therefore result in gradual A+T-enrichment of the genic remnants already released from amino-acid-coding constraints in recent past. The base composition of such remnants is expected to gradually approach the A+T-content of *bona fide *non-coding regions.

Comparison of orthologous gene synteny from five representative strains having different genome size and G+C-content clearly points at a high level of chromosomal rearrangement during genome shrinkage in *Prochlorococcus*. This finding is in agreement with earlier findings on association of chromosomal rearrangement events with higher rates of chromosomal evolution and/or the phenomenon of genome reduction, as in *Arabidopsis thaliana *[40] and different endoparasites/endosymbionts [41,42]. Intra-chromosomal recombination at duplicated sequences often results in deletion of intervening sequences, and rearrangement of flanking regions, thereby leading to genome shrinkage [39].

Previous analyses with endosymbiotic or endoparasitic organisms like *Bartonella*, *Tropheryma*, *Buchnera*, *Wigglesworthia *etc. [2,3,28,29] revealed that the phenomenon of genome reduction is normally associated with population bottlenecks or other mechanisms such as selective sweeps. In case of the hyperthermophile *Nanoarchaeum equitans*, extreme genome reduction is a feature of its thermoparasitic adaptation [1]. Although our knowledge of bacterial populations in open oceans is not exhaustive, it may certainly be assumed that *P. marinus *ecotypes, the most abundant free-living marine cyanobacteria and an important contributor to global photosynthesis, are not subject to small population sizes [13-15,30]. More importantly, the HL strains with reduced genomes are apparently biologically superior than their LL counterparts [21]. It is possible that the bias towards reduced A+T rich genomes in HL strains is consistent with cellular economy at regions with limited nitrogen and phosphorous near the ocean surface. Scarcity of these elements that are essential in DNA synthesis favors the incorporation of an AT base-pair containing seven atoms of nitrogen, one less than a GC base-pair. It is worth mentioning at this point that the trends in amino acid usage in different *P. marinus *strains, as observed in this study are quite compatible with the earlier report by Lv *et al*. [43] on influence of resource availability on proteome composition of these species. For instance, increase in overall aromaticity from LLa to LLb and HL strains is in full agreement with the observations by Lv *et al*. [43] on increased carbon-content in the encoded proteins of different HL strains as compared to that of LL strains. The average instability indices of the HL proteins are significantly lower than those of their LL orthologs, suggesting that the HL proteins, in general, may be more stable. Proteins characterized by higher percentages of helix structures, experience increased overall packing that imparts more rigidity [44] and, hence, a decrease in regions with helix-forming propensities with a subsequent increase in coiled structures in HL proteins probably makes them more flexible. It is also tempting to presume that higher values of aromaticity and pI in HL proteins, as compared to LL orthologs, might facilitate cation-pi interactions in the former, imparting more stability. The central issue in the adaptation of HL proteins to their environmental niches may, therefore, be the conservation of their functional state, characterized by a well-balanced optimization of stability and flexibility.

## Conclusion

The current study advocates for the presence of adaptive selection forces that might have played significant role in governing *Prochlorococcus *evolution and fitness at the genome and proteome levels. An optimization between these adaptive forces and directional mutational bias has set definite trends in molecular evolution of *P. marinus*. This characterizes different *P. marinus *ecotypes with distinct niche-specific compositional, physicochemical and structural traits, thereby driving them chronologically towards increasing stability and/or fidelity.

## Methods

### Sequence retrieval

All predicted protein coding sequences and the complete genome sequences of the 12 different strains of *P. marinus *were retrieved from the NCBI GenBank (listed in Table 1). For comparison, the predicted protein coding sequences of *E. coli *(NC_000913.2), *Bacillus cereus *(NC_003909.8), *Francisella tularensis *(NC_006570.1), *Synechococcus elongatus *(NC_006576.1), *Synechocystis sp*. (NC_000911.1), *Nostoc sp*. (NC_003272.1), *Campylobacter jejuni *(NC_003912.7), *Cyanothece *(NC_011884.1) and *Gramella forsetii *(NC_008571.1) were also retrieved from GenBank. Annotated ORFs, which encode proteins less than 100 amino acids long, were not considered for further analysis.

### Determination of leading and lagging strand genes

In order to identify the replication origin (oriC) or termination (ter) sites we performed GC-skew (G-C/G+C) analysis using a sliding window of 10 Kb along the genome sequence. The sites were validated by checking the neighbouring gene organization (*e.g*. identified origins in *Prochlorococcus *genomes were flanked by DNA polymerase beta subunit III gene on the 3' side and the Threonine synthatase gene on the 5' side) and the presence of DnaA boxes in their vicinity [45]. Based on the predicted oriC and ter sites (Additional file 6), the leading strands and lagging strands of replication for each genomes were identified along with the genes encoded on the two strands.

### Multivariate analyses on synonymous codon and amino acid usage and cluster analysis on amino acid usage

Correspondence analysis (COA) on relative synonymous codon usage (RSCU) and amino acid usage of genes/proteins were performed on individual genomes in order to identify any significant variation in the usage of codons or amino acids, if present, and help ascertain the underlying cause(s), using the program CODONW 1.4.2 [46].

To find out the variation in amino acid usage between LL and HL *Prochlorococcus *strains, a cluster analysis on standardized amino acid usage was carried out using STATISTICA (version 6.0, published by Statsoft Inc., USA) for all 12 *Prochlorococcus *organisms (Table 1) along with *E. coli*, *Cyanothece*, *C. jejuni *and *G. forsetii *having G+C-content nearly equal to the different LL and HL strains. The amino acid usage of *E. coli *was chosen as a well-defined reference for standardizing the amino acid composition for the analysis and to produce an accompanying heat map. With the help of a program developed in-house in Visual Basic, a 16 × 20 matrix (heatmap) was generated, where the rows and the columns correspond to data sources (*i.e*., organisms in the cluster) and standardized amino acid usage values, respectively. The overrepresentation or underrepresentation of standardized amino acid usage values of the organisms in the matrix are shown in green or red colored blocks (Figure 4) respectively, and their intensities varying in accordance with their deviation from the standard (yellow). The extreme left column represents the genomic G+C-content of the respective organisms.

### Dinucleotide analysis of DNA sequences

For all *Prochlorococcus *genomes and *E. coli*, the dinucleotide abundance for each possible dinucleotide was calculated as the ratio between the observed and expected frequencies of the concerned dinucleotide in its genomic context [47]. Dinucleotide abundance values generally represent the genomic signature of any species [48] and here we were interested to see whether all *Prochlorococcus *genomes follow a similar trend or not.

### Determination of orthologs

Stand alone BLAST package (ver. 2.2.18) was downloaded from the NCBI FTP site and using the package, all-to-all BLASTN and BLASTP searches were performed with the genes from all the 12 strains of *P. marinus*. Orthologs across these organisms were defined for this study as protein coding genes having a BLASTP sequence Identity ≥ 60%, not more than 20% difference in length and E-value ≤ 1e-20. The resultant list of 'orthologs' were checked for consistency with the data obtained from Genplot http://www.ncbi.nlm.nih.gov/sutils/geneplot.cgi, which houses a pair-wise list of genes giving mutually best BLASTP hits when all genes from the genomes of any two organisms are 'blasted' against each other. We have identified 519 orthologs present in all 12 *P. marinus *genomes as their core proteome. The stringent measures employed for the similarity search ensure that these orthologs have been sufficiently conserved throughout the adaptive evolution of *P. marinus*, and any niche-specific features deciphered from this dataset would certainly not be a trivial outcome. For comparative analysis with suitable outgroup organisms, we retrieved orthologs of nine organisms including three representative *P. marinus *strains (LL1, LL3 and HL3), *E. coli*, *B. cereus*, *F. tularensis*, *S. elongatus*, *Synechocystis sp*. and *Nostoc sp*. from NCBI GenePlot by filtering the symmetrical best hits of protein homologs.

### Estimation of synonymous and non-synonymous substitution patterns in orthologous sequences

Positive selection can be inferred from a higher proportion of non-synonymous over synonymous substitutions per site (d_N_/d_S _> 1). The d_N _and d_S _values were calculated for 519 orthologs of LL1, LL6 and HL3 using the software MEGA (version 4) [49]. The calculation was based on the modified Nei-Gojobori Jukes-Cantor method that considers deviations from an equal frequency of transitions and transversions [50,51].

### Gene synteny visualization

Comparison of the gene repertoire or gene synteny between 5 representative *Prochlorococcus *strains (LL1, LL3, LL6, HL3 and HL4) were carried out using a Java program developed in-house. It can represent the arrangement of orthologous genes between two chromosomes by joining the locations of the orthologs by differently coloured lines. The red lines represent the genes present on the same strand (+/-) and blue lines represent orthologs coded on different strands of chromosomes being compared.

### Calculation of codon/amino acid usage indices and estimation of secondary structure of proteins

Indices like relative synonymous codon usage (RSCU) [52], G+C and G+T-content at third codon positions (GC3 & GT3 respectively), aromaticity and average hydrophobicity (Gravy score) [53] of protein coding sequences were calculated to find out the factors influencing codon and amino acid usages. The isoelectric point (pI) and instability index [54] of each protein were calculated using the Expasy proteomics server [55]. Secondary structures of the identified orthologs were computed using the software PREDATOR [56] and the varying percentages of the structural components (*viz*. helices, sheets, and coils) in proteins from different strains were also noted.

### Identification of intergenic regions

The sequences coding for mRNAs and structural RNAs were noted from the protein table and structural RNA table respectively (available from NCBI) for each of the organisms. Intergenic regions were identified by subtracting the regions of these gene sequences from the whole genome. The overall G+C-content of the intergenic regions were calculated after concatenating all the intergenic sequences together, for each of the 12 *Prochlorococcus *strains. For identification of probable pseudogenes/remnants of coding DNA in LL6 and HL3 (two representative strains of groups LLb and HL having reduced genomes), their intergenic regions were subjected to a similarity search (tBlastX) against a pool of *Prochlorococcal *genes (consisting of sequences from three representative strains LL1, LL6, HL3). 48 hits for LL6 and 93 hits for HL3 were identified, having sequence identities ≥ 30%, aligned lengths ≥ 15 amino acids, and E-values < 1e-3.

## Abbreviations

COA: correspondence analysis; LL: low light; HL: high light; pI: isoelectric point; RSCU: relative synonymous codon usage; GT3: G+T-content at third codon positions; GC3: G+C-content at third codon positions.

## Authors' contributions

SP and AD made substantial contributions to the design of the study, devised and carried out the overall strategy and drafted the manuscript. SKB developed relevant programs for data mining and analysis of genome sequences and also participated in sequence analysis. SD participated in the initial phase of the work, made thoughtful discussion during execution of the project and preparation of the manuscript. CD conceived and coordinated the study and revised the manuscript critically for important intellectual content. All authors read and approved the final manuscript.

## Supplementary Material

Additional file 1 Relative Synonymous Codon Usage of leading and lagging strand genes of *P. marinus *str. MIT9313 (LL1). Click here for file

Additional file 2 Relative Synonymous Codon Usage of leading and lagging strand genes of *P. marinus *str. NATL2A (LL6). Click here for file

Additional file 3 Dinucleotide abundance values of twelve *P. marinus *genomes and *E. coli*. Click here for file

Additional file 4 Average G+C-contents (%) of the overall genome, coding sequences and intergenic sequences of 12 *Prochlorococcus *strains. Click here for file

Additional file 5 List of putative remnants of coding regions in LL6 and HL3. Click here for file

Additional file 6 Predicted locations of origins and termini of replication of the 12 *Prochlorococcus *strains. Click here for file

## References

[B1] Das S Paul S Bag SK Dutta C Analysis of Nanoarchaeum equitans genome and proteome composition: indications for hyperthermophilic and parasitic adaptation BMC Genomics 2006 7 186 10.1186/1471-2164-7-186 16869956 PMC1574309

[B2] Das S Paul S Chatterjee S Dutta C Codon and amino acid usage in two major human pathogens of genus Bartonella--optimization between replicational-transcriptional selection, translational control and cost minimization DNA Res 2005 12 2 91 102 10.1093/dnares/12.2.91 16303741

[B3] Das S Paul S Dutta C Evolutionary constraints on codon and amino acid usage in two strains of human pathogenic actinobacteria Tropheryma whipplei J Mol Evol 2006 62 5 645 658 10.1007/s00239-005-0164-6 16557339

[B4] Eisenberg H Life in unusual environments: progress in understanding the structure and function of enzymes from extreme halophilic bacteria Arch Biochem Biophys 1995 318 1 1 5 10.1006/abbi.1995.1196 7726549

[B5] Moran NA Wernegreen JJ Lifestyle evolution in symbiotic bacteria: insights from genomics Trends Ecol Evol 2000 15 8 321 326 10.1016/S0169-5347(00)01902-9 10884696

[B6] Paul S Bag SK Das S Harvill ET Dutta C Molecular signature of hypersaline adaptation: insights from genome and proteome composition of halophilic prokaryotes Genome Biol 2008 9 4 R70 10.1186/gb-2008-9-4-r70 18397532 PMC2643941

[B7] Pikuta EV Hoover RB Tang J Microbial extremophiles at the limits of life Crit Rev Microbiol 2007 33 3 183 209 10.1080/10408410701451948 17653987

[B8] Singer GA Hickey DA Thermophilic prokaryotes have characteristic patterns of codon usage, amino acid composition and nucleotide content Gene 2003 317 1-2 39 47 10.1016/S0378-1119(03)00660-7 14604790

[B9] Bliska JB Casadevall A Intracellular pathogenic bacteria and fungi--a case of convergent evolution? Nat Rev Microbiol 2009 7 2 165 171 19098923 10.1038/nrmicro2049

[B10] Merhej V Royer-Carenzi M Pontarotti P Raoult D Massive comparative genomic analysis reveals convergent evolution of specialized bacteria Biol Direct 2009 4 13 10.1186/1745-6150-4-13 19361336 PMC2688493

[B11] Mongodin EF Nelson KE Daugherty S Deboy RT Wister J Khouri H Weidman J Walsh DA Papke RT Sanchez Perez G The genome of Salinibacter ruber: convergence and gene exchange among hyperhalophilic bacteria and archaea Proc Natl Acad Sci USA 2005 102 50 18147 18152 10.1073/pnas.0509073102 16330755 PMC1312414

[B12] Chisholm S Olson R Zettler E Goericke R Waterbury J Welschmeyer N A novel free-living prochlorophyte abundant in the oceanic euphotic zone Nature 1988 334 340 343 10.1038/334340a0

[B13] Goericke R Welschmeyer N The marine prochlorophyte Prochlorococcus contributes significantly to phytoplankton biomass and primary production in the Sargasso Sea Deep-sea research Part 1 Oceanographic research papers 1993 40 11-12 2283 2294 10.1016/0967-0637(93)90104-B

[B14] Partensky F Blanchot J Vaulot D Differential distribution and ecology of Prochlorococcus and Synechococcus in oceanic waters: a review Bulletin de l'Institut océanographique(Monaco) 1999 457 475

[B15] Partensky F Hess WR Vaulot D Prochlorococcus, a marine photosynthetic prokaryote of global significance Microbiol Mol Biol Rev 1999 63 1 106 127 10066832 10.1128/mmbr.63.1.106-127.1999 PMC98958

[B16] Moore LR Rocap G Chisholm SW Physiology and molecular phylogeny of coexisting Prochlorococcus ecotypes Nature 1998 393 6684 464 467 10.1038/30861 9624000

[B17] Urbach E Scanlan DJ Distel DL Waterbury JB Chisholm SW Rapid diversification of marine picophytoplankton with dissimilar light-harvesting structures inferred from sequences of Prochlorococcus and Synechococcus (Cyanobacteria) J Mol Evol 1998 46 2 188 201 10.1007/PL00006294 9452521

[B18] West NJ Scanlan DJ Niche-partitioning of Prochlorococcus populations in a stratified water column in the eastern North Atlantic Ocean Appl Environ Microbiol 1999 65 6 2585 2591 10347047 10.1128/aem.65.6.2585-2591.1999 PMC91382

[B19] Kettler GC Martiny AC Huang K Zucker J Coleman ML Rodrigue S Chen F Lapidus A Ferriera S Johnson J Patterns and implications of gene gain and loss in the evolution of Prochlorococcus PLoS Genet 2007 3 12 e231 10.1371/journal.pgen.0030231 18159947 PMC2151091

[B20] Rocap G Larimer FW Lamerdin J Malfatti S Chain P Ahlgren NA Arellano A Coleman M Hauser L Hess WR Genome divergence in two Prochlorococcus ecotypes reflects oceanic niche differentiation Nature 2003 424 6952 1042 1047 10.1038/nature01947 12917642

[B21] Coleman ML Chisholm SW Code and context: Prochlorococcus as a model for cross-scale biology Trends Microbiol 2007 15 9 398 407 10.1016/j.tim.2007.07.001 17693088

[B22] Garcia-Fernandez JM Diez J Adaptive mechanisms of nitrogen and carbon assimilatory pathways in the marine cyanobacteria Prochlorococcus Res Microbiol 2004 155 10 795 802 10.1016/j.resmic.2004.06.009 15567272

[B23] Martiny AC Coleman ML Chisholm SW Phosphate acquisition genes in Prochlorococcus ecotypes: evidence for genome-wide adaptation Proc Natl Acad Sci USA 2006 103 33 12552 12557 10.1073/pnas.0601301103 16895994 PMC1567916

[B24] Sullivan MB Waterbury JB Chisholm SW Cyanophages infecting the oceanic cyanobacterium Prochlorococcus Nature 2003 424 6952 1047 1051 10.1038/nature01929 12944965

[B25] Dufresne A Garczarek L Partensky F Accelerated evolution associated with genome reduction in a free-living prokaryote Genome Biol 2005 6 2 R14 10.1186/gb-2005-6-2-r14 15693943 PMC551534

[B26] Berg OG Kurland CG Evolution of microbial genomes: sequence acquisition and loss Mol Biol Evol 2002 19 12 2265 2276 12446817 10.1093/oxfordjournals.molbev.a004050

[B27] Lynch M Blanchard JL Deleterious mutation accumulation in organelle genomes Genetica 1998 102-103 1-6 29 39 10.1023/A:1017022522486 9720269

[B28] Wernegreen JJ Genome evolution in bacterial endosymbionts of insects Nat Rev Genet 2002 3 11 850 861 10.1038/nrg931 12415315

[B29] Wernegreen JJ Moran NA Evidence for genetic drift in endosymbionts (Buchnera): analyses of protein-coding genes Mol Biol Evol 1999 16 1 83 97 10331254 10.1093/oxfordjournals.molbev.a026040

[B30] Hu J Blanchard JL Environmental sequence data from the Sargasso Sea reveal that the characteristics of genome reduction in Prochlorococcus are not a harbinger for an escalation in genetic drift Mol Biol Evol 2009 26 1 5 13 10.1093/molbev/msn217 18845550

[B31] Garcia-Fernandez JM de Marsac NT Diez J Streamlined regulation and gene loss as adaptive mechanisms in Prochlorococcus for optimized nitrogen utilization in oligotrophic environments Microbiol Mol Biol Rev 2004 68 4 630 638 10.1128/MMBR.68.4.630-638.2004 15590777 PMC539009

[B32] Dufresne A Salanoubat M Partensky F Artiguenave F Axmann IM Barbe V Duprat S Galperin MY Koonin EV Le Gall F Genome sequence of the cyanobacterium Prochlorococcus marinus SS120, a nearly minimal oxyphototrophic genome Proc Natl Acad Sci USA 2003 100 17 10020 10025 10.1073/pnas.1733211100 12917486 PMC187748

[B33] Marais GA Calteau A Tenaillon O Mutation rate and genome reduction in endosymbiotic and free-living bacteria Genetica 2008 134 2 205 210 10.1007/s10709-007-9226-6 18046510

[B34] Lafay B Lloyd AT McLean MJ Devine KM Sharp PM Wolfe KH Proteome composition and codon usage in spirochaetes: species-specific and DNA strand-specific mutational biases Nucleic Acids Res 1999 27 7 1642 1649 10.1093/nar/27.7.1642 10075995 PMC148367

[B35] McInerney JO Replicational and transcriptional selection on codon usage in Borrelia burgdorferi Proc Natl Acad Sci USA 1998 95 18 10698 10703 10.1073/pnas.95.18.10698 9724767 PMC27958

[B36] Gentles AJ Karlin S Genome-scale compositional comparisons in eukaryotes Genome Res 2001 11 4 540 546 10.1101/gr.163101 11282969 PMC311039

[B37] Michaels ML Cruz C Grollman AP Miller JH Evidence that MutY and MutM combine to prevent mutations by an oxidatively damaged form of guanine in DNA Proc Natl Acad Sci USA 1992 89 15 7022 7025 10.1073/pnas.89.15.7022 1495996 PMC49637

[B38] Horst JP Wu TH Marinus MG Escherichia coli mutator genes Trends Microbiol 1999 7 1 29 36 10.1016/S0966-842X(98)01424-3 10068995

[B39] Andersson SG Zomorodipour A Andersson JO Sicheritz-Ponten T Alsmark UC Podowski RM Naslund AK Eriksson AS Winkler HH Kurland CG The genome sequence of Rickettsia prowazekii and the origin of mitochondria Nature 1998 396 6707 133 140 10.1038/24094 9823893

[B40] Yogeeswaran K Frary A York TL Amenta A Lesser AH Nasrallah JB Tanksley SD Nasrallah ME Comparative genome analyses of Arabidopsis spp.: inferring chromosomal rearrangement events in the evolutionary history of A. thaliana Genome Res 2005 15 4 505 515 10.1101/gr.3436305 15805492 PMC1074365

[B41] Belda E Moya A Silva FJ Genome rearrangement distances and gene order phylogeny in gamma-Proteobacteria Mol Biol Evol 2005 22 6 1456 1467 10.1093/molbev/msi134 15772379

[B42] Mira A Ochman H Moran NA Deletional bias and the evolution of bacterial genomes Trends Genet 2001 17 10 589 596 10.1016/S0168-9525(01)02447-7 11585665

[B43] Lv J Li N Niu DK Association between the availability of environmental resources and the atomic composition of organismal proteomes: evidence from Prochlorococcus strains living at different depths Biochem Biophys Res Commun 2008 375 2 241 246 10.1016/j.bbrc.2008.08.011 18706891

[B44] Fleming PJ Richards FM Protein packing: dependence on protein size, secondary structure and amino acid composition J Mol Biol 2000 299 2 487 498 10.1006/jmbi.2000.3750 10860754

[B45] Mackiewicz P Zakrzewska-Czerwinska J Zawilak A Dudek MR Cebrat S Where does bacterial replication start? Rules for predicting the oriC region Nucleic Acids Res 2004 32 13 3781 3791 10.1093/nar/gkh699 15258248 PMC506792

[B46] Penden J Analysis of codon usage PhD thesis 1997 University of Nottingham, Department of Genetics

[B47] Karlin S Burge C Dinucleotide relative abundance extremes: a genomic signature Trends Genet 1995 11 7 283 290 10.1016/S0168-9525(00)89076-9 7482779

[B48] Karlin S Mrazek J Campbell AM Compositional biases of bacterial genomes and evolutionary implications J Bacteriol 1997 179 12 3899 3913 9190805 10.1128/jb.179.12.3899-3913.1997 PMC179198

[B49] Tamura K Dudley J Nei M Kumar S MEGA4: Molecular Evolutionary Genetics Analysis (MEGA) software version 4.0 Mol Biol Evol 2007 24 8 1596 1599 10.1093/molbev/msm092 17488738

[B50] Nei M Gojobori T Simple methods for estimating the numbers of synonymous and nonsynonymous nucleotide substitutions Mol Biol Evol 1986 3 5 418 426 3444411 10.1093/oxfordjournals.molbev.a040410

[B51] Nei M Kumar S Molecular evolution and phylogenetics 2000 Oxford University Press, USA

[B52] Sharp PM Li WH The codon Adaptation Index--a measure of directional synonymous codon usage bias, and its potential applications Nucleic Acids Res 1987 15 3 1281 1295 10.1093/nar/15.3.1281 3547335 PMC340524

[B53] Kyte J Doolittle RF A simple method for displaying the hydropathic character of a protein J Mol Biol 1982 157 1 105 132 10.1016/0022-2836(82)90515-0 7108955

[B54] Guruprasad K Reddy BV Pandit MW Correlation between stability of a protein and its dipeptide composition: a novel approach for predicting in vivo stability of a protein from its primary sequence Protein Eng 1990 4 2 155 161 10.1093/protein/4.2.155 2075190

[B55] Expasy Proteomics Server http://expasy.org

[B56] Frishman D Argos P Seventy-five percent accuracy in protein secondary structure prediction Proteins 1997 27 3 329 335 10.1002/(SICI)1097-0134(199703)27:3<329::AID-PROT1>3.0.CO;2-8 9094735

