# Supplementary material for: Distinct, ecotype-specific genome and proteome signatures in the marine cyanobacteria Prochlorococcus
Source: BMC Genomics. 2010 Feb 10;11:103. doi: 10.1186/1471-2164-11-103 (PMC2836286; doi:10.1186/1471-2164-11-103)
Supplement: Additional file 1 — Relative Synonymous Codon Usage of leading and lagging strand genes of P. marinus str. MIT9313 (LL1). [file 1471-2164-11-103-S1.PDF]

Additional file 1: Relative Synonymous Codon Usage of leading and lagging strand genes of *P. marinus* str. MIT9313 (LL1)

Amino Acid	Codon	Leading strand	Lagging strand	Amino Acid	Codon	Leading strand	Lagging strand
		RSCU	RSCU			RSCU	RSCU
Phe	UUU	1.22 *	0.90	Tyr	UAU	1.27 *	0.99
	UUC	0.78	1.10*		UAC	0.73	1.01 *
Leu	UUA	0.49	0.49	TER	UAA	0.74	1.08
	UUG	1.71 *	0.81		UAG	0.44	0.34
	CUU	1.37 *	1.12	His	CAU	1.38 *	1.12
	CUC	0.82	1.25 *		CAC	0.62	0.88 *
	CUA	0.39	0.82 *	Gln	CAA	0.74	1.19 *
	CUG	1.22	1.51 *		CAG	1.26 *	0.81
Ile	AUU	1.46 *	1.05	Asn	AAU	1.24 *	0.95
	AUC	1.28	1.68 *		AAC	0.76	1.05 *
	AUA	0.25	0.27	Lys	AAA	0.79	1.10 *
Met	AUG	1.00	1.00		AAG	1.21 *	0.90
Val	GUU	1.30 *	0.99	Asp	GAU	1.45 *	1.18
	GUC	0.66	0.95 *		GAC	0.55	0.82 *
	GUA	0.38	0.61 *	Glu	GAA	0.88	1.19 *
	GUG	1.66 *	1.46		GAG	1.12 *	0.81
Ser	UCU	1.19 *	0.76	Cys	UGU	1.05 *	0.70
	UCC	0.67	0.83 *		UGC	0.95	1.3 *
	UCA	0.90	1.05 *	TER	UGA	1.83	1.58
	UCG	0.65 *	0.44	Trp	UGG	1.00	1.00
Pro	CCU	1.47 *	1.05	Arg	CGU	1.67 *	1.17
	CCC	0.76	1.05 *		CGC	1.57	2.04 *
	CCA	1.05	1.39 *		CGA	0.85	1.06 *
	CCG	0.72 *	0.51		CGG	0.80 *	0.57
Thr	ACU	1.15 *	0.82	Ser	AGU	1.28 *	0.95
	ACC	1.22	1.52 *		AGC	1.31	1.97 *
	ACA	0.86	1.13 *	Arg	AGA	0.49	0.66 *
	ACG	0.78 *	0.53		AGG	0.62 *	0.50
Ala	GCU	1.43 *	1.00	Gly	GGU	1.34 *	0.96
	GCC	1.03	1.49 *		GGC	1.21	1.56 *
	GCA	0.88	1.07 *		GGA	0.79	0.91 *
	GCG	0.66 *	0.44		GGG	0.66 *	0.58

Note: * indicates corresponding codons are significantly over-expressed among the genes encoded on the leading strand or the lagging strand ($p<10^{-3}$).
