# Supplementary material for: Distinct, ecotype-specific genome and proteome signatures in the marine cyanobacteria Prochlorococcus
Source: BMC Genomics. 2010 Feb 10;11:103. doi: 10.1186/1471-2164-11-103 (PMC2836286; doi:10.1186/1471-2164-11-103)
Supplement: Additional file 2 — Relative Synonymous Codon Usage of leading and lagging strand genes of P. marinus str. NATL2A (LL6). [file 1471-2164-11-103-S2.PDF]

Additional file 2: Relative Synonymous Codon Usage of leading and lagging strand genes of *P. marinus* str. NATL2A (LL6)

Amino Acid	Codon	Leading strand	Lagging strand	Amino Acid	Codon	Leading strand	Lagging strand
		RSCU	RSCU			RSCU	RSCU
Phe	UUU	1.60 *	1.44	Tyr	UAU	1.58 *	1.45
	UUC	0.40	0.56 *		UAC	0.42	0.55 *
Leu	UUA	2.22	2.34 *	TER	UAA	1.65	2.00
	UUG	1.26 *	0.60		UAG	0.55	0.48
	CUU	1.44	1.50	His	CAU	1.62 *	1.5
	CUC	0.26	0.37 *		CAC	0.38	0.50 *
	CUA	0.59	0.96 *	Gln	CAA	1.52	1.69 *
	CUG	0.22	0.24		CAG	0.48 *	0.31
Ile	AUU	1.73 *	1.45	Asn	AAU	1.65 *	1.56
	AUC	0.41	0.49 *		AAC	0.35	0.44 *
	AUA	0.87	1.07 *	Lys	AAA	1.48	1.59 *
Met	AUG	1.00	1.00		AAG	0.52 *	0.41
Val	GUU	2.14 *	1.87	Asp	GAU	1.67 *	1.53
	GUC	0.49	0.56 *		GAC	0.33	0.47 *
	GUA	0.97	1.25 *	Glu	GAA	1.37	1.5 0*
	GUG	0.41 *	0.32		GAG	0.63 *	0.50
Ser	UCU	1.92 *	1.41	Cys	UGU	1.41 *	1.19
	UCC	0.36	0.40		UGC	0.59	0.81 *
	UCA	1.56	1.86 *	TER	UGA	0.80	0.51
	UCG	0.29 *	0.23	Trp	UGG	1.00	1.00
Pro	CCU	1.84 *	1.58	Arg	CGU	0.83 *	0.63
	CCC	0.35	0.42 *		CGC	0.29	0.33
	CCA	1.61	1.80 *		CGA	0.74	0.66
	CCG	0.21	0.21		CGG	0.13	0.10
Thr	ACU	1.90 *	1.59	Ser	AGU	1.37	1.40
	ACC	0.44	0.48		AGC	0.49	0.71 *
	ACA	1.34	1.65 *	Arg	AGA	3.08	3.49 *
	ACG	0.32	0.27		AGG	0.93 *	0.80
Ala	GCU	1.79 *	1.52	Gly	GGU	1.44 *	1.27
	GCC	0.40	0.51 *		GGC	0.53	0.52
	GCA	1.46	1.68 *		GGA	1.58	1.71 *
	GCG	0.35 *	0.30		GGG	0.46	0.50

Note: *indicates corresponding codons are significantly over-expressed among the genes encoded on the leading strand or the lagging strand ($p<10^{-3}$).
