# Supplementary material for: Distinct, ecotype-specific genome and proteome signatures in the marine cyanobacteria Prochlorococcus
Source: BMC Genomics. 2010 Feb 10;11:103. doi: 10.1186/1471-2164-11-103 (PMC2836286; doi:10.1186/1471-2164-11-103)
Supplement: Additional file 3 — Dinucleotide abundance values of twelve P. marinus genomes and E. coli. [file 1471-2164-11-103-S3.PDF]

Additional file 3: Dinucleotide abundance values of 12 *P. marinus* genomes and *E. coli*. Significantly overrepresented dinucleotide abundance values are indicated in red when an abundance value is ≥ 1.23, while significantly underrepresentation is shown in blue when an abundance value is ≤ 0.78 (in bold face if ≤ 0.70) (as described by Karlin *et al.*, *Theoretical population biology*, 61, 367-390, 2002).

Organism	Dinucleotide Abundances									
	AA TT	AC GT	AG CT	AT	CA TG	CC GG	CG	GA TC	GC	TA
**LL1**	1.14	0.78	1.08	1.00	1.24	0.95	0.74	1.04	1.22	**0.57**
**LL2**	1.14	0.78	1.07	1.01	1.23	0.96	0.74	1.04	1.22	**0.60**
**LL3**	1.15	0.73	1.14	0.92	1.07	1.10	**0.52**	1.06	1.27	0.78
**LL4**	1.15	0.74	1.16	0.91	1.07	1.11	**0.52**	1.05	1.23	0.78
**LL5**	1.16	0.74	1.11	0.92	1.06	1.13	**0.57**	1.09	1.17	0.76
**LL6**	1.17	0.74	1.11	0.92	1.06	1.13	**0.57**	1.09	1.18	0.75
**HL1**	1.18	0.72	1.08	0.91	1.02	1.26	**0.51**	1.08	1.18	0.77
**HL2**	1.17	0.71	1.09	0.92	1.02	1.26	**0.49**	1.08	1.19	0.78
**HL3**	1.17	0.72	1.09	0.92	1.01	1.28	**0.51**	1.08	1.17	0.79
**HL4**	1.17	0.72	1.08	0.92	1.01	1.28	**0.52**	1.09	1.16	0.79
**HL5**	1.18	0.71	1.08	0.91	1.02	1.26	**0.51**	1.09	1.19	0.77
**HL6**	1.18	0.72	1.08	0.91	1.02	1.25	**0.51**	1.08	1.18	0.77
***E. coli* K12**	1.21	0.88	0.82	1.10	1.12	0.90	1.15	0.92	1.28	0.75
