# Supplementary material for: Distinct, ecotype-specific genome and proteome signatures in the marine cyanobacteria Prochlorococcus
Source: BMC Genomics. 2010 Feb 10;11:103. doi: 10.1186/1471-2164-11-103 (PMC2836286; doi:10.1186/1471-2164-11-103)
Supplement: Additional file 4 — Average G+C-contents (%) of the overall genome, coding sequences and intergenic sequences of 12 Prochlorococcus strains. [file 1471-2164-11-103-S4.PDF]

**Additional file 4:** G+C-content (%) of the overall genome, coding sequences and intergenic sequences of 12 *Prochlorococcus* strains.

| Organism | Genomic G+C% | Coding G+C% | Intergenic G+C% |
|---|---|---|---|
| **LL1** | 50.74 | 52.17 | 43.97 |
| **LL2** | 50.01 | 51.25 | 43.09 |
| **LL3** | 36.44 | 37.04 | 30.68 |
| **LL4** | 38.01 | 38.57 | 32.21 |
| **LL5** | 34.98 | 35.79 | 28.81 |
| **LL6** | 35.12 | 35.98 | 28.76 |
| **HL1** | 31.32 | 32.03 | 22.83 |
| **HL2** | 31.21 | 31.97 | 23.63 |
| **HL3** | 30.80 | 31.64 | 23.33 |
| **HL4** | 30.79 | 31.68 | 22.68 |
| **HL5** | 31.15 | 31.91 | 23.53 |
| **HL6** | 31.34 | 32.06 | 22.59 |
