# Supplementary material for: Distinct, ecotype-specific genome and proteome signatures in the marine cyanobacteria Prochlorococcus
Source: BMC Genomics. 2010 Feb 10;11:103. doi: 10.1186/1471-2164-11-103 (PMC2836286; doi:10.1186/1471-2164-11-103)
Supplement: Additional file 6 — Predicted locations of origins and termini of replication of the 12 Prochlorococcus strains. [file 1471-2164-11-103-S6.PDF]

Additional file 6: Predicted locations of origins and termini of replication of the 12 *Prochlorococcus* strains.

Abbr.	Organism	Origin (bp)	Terminus (bp)
**LL1**	*P. marinus* str. MIT 9313	0	795000
**LL2**	*P. marinus* str. MIT 9303	0	1083300
**LL3**	*P. marinus* subsp. marinus str. CCMP1375 (SS120)	0	869800
**LL4**	*P. marinus* str. MIT 9211	0	740000
**LL5**	*P. marinus* str. NATL1A	0	1070200
**LL6**	*P. marinus* str. NATL2A	1292490	335400
**HL1**	*P. marinus* str. AS9601	0	976600
**HL2**	*P. marinus* str. MIT 9312	0	857250
**HL3**	*P. marinus* subsp. pastoris str. CCMP1986 (MED 4)	0	855300
**HL4**	*P. marinus* str. MIT 9515	0	724500
**HL5**	*P. marinus* str. MIT 9215	0	980000
**HL6**	*P. marinus* str. MIT 9301	0	800000
